# From bugs to behavior: targeting the gut-brain interface for addiction therapeutics

**DOI:** 10.1080/19490976.2025.2569740

**Published:** 2025-10-28

**Authors:** Yiping Ru, Jie Jia, Xiyao Yang, Xuejiao Ba, Jiacong Yan, Hongqing Zhang, Yizhi Zhang

**Affiliations:** a NHC Key Laboratory of Drug Addiction Medicine, Kunming Medical University, Kunming, Yunnan, China; b Charité-Universitätsmedizin Berlin, Berlin, Germany; c Scientific Research Laboratory Center, First Affiliated Hospital of Kunming Medical University, Kunming, Yunnan, China; d Obstetrics and Gynecology Clinical Key Laboratory, Kunming Women and Children's Health Hospital, Kunming, Yunnan, China; e Department of Reproductive Medicine, The First People's Hospital of Yunnan Province, The Affiliated Hospital of Kunming University of Science and Technology, NHC Key Laboratory of Preconception Health Birth in Western China, Kunming, Yunnan, China

**Keywords:** Substance use disorder, intestinal microbiota, alcohol, cocaine, nicotine, opioids, methamphetamine, therapeutic interventions

## Abstract

Substance use disorders involving alcohol, cocaine, tobacco, opioids, and methamphetamine represent a significant global health burden, driven by the dysregulation of neurobiological and metabolic pathways in addiction. Emerging research highlights the gut-brain axis (GBA) as a critical mediator of addictive behaviors, with gut microbiota and their metabolites influencing bidirectional communication between the gastrointestinal tract and the central nervous system. This review synthesizes current evidence on how gut microbial communities modulate the brain’s reward circuitry and the metabolism of addictive substances, thereby shaping behavioral and physiological responses to substance use. We explore the mechanistic interplay between microbial-derived metabolites (*e.g.,* short-chain fatty acids, neurotransmitters) and host neuroimmune signaling, which may reinforce compulsive drug-seeking behaviors. Additionally, we discuss how chronic substance exposure alters gut microbiota composition and intestinal barrier integrity, perpetuating a vicious cycle of addiction. By reframing addiction through the lens of the GBA, this review highlights the therapeutic potential of microbiota-targeted interventions, such as probiotics, prebiotics, and dietary modulation, to restore gut-brain homeostasis and mitigate relapse. These insights advocate for a paradigm shift in understanding addiction as a systemic disorder, offering novel avenues for biomarker discovery and personalized treatment strategies.

## Introduction

According to the Diagnostic and Statistical Manual of Mental Disorders, Fifth Edition (DSM−5), substance use disorder (SUD) refers to the harmful use of psychoactive substances, which include alcohol, caffeine, tobacco, cannabis, opioids, hallucinogens, inhalants, sedatives, hypnotics, and anxiolytics. SUD is linked to a range of adverse health effects, notably impairments in brain function and the development of maladaptive behaviors. These behaviors often involve the escalation of tolerance, withdrawal symptoms, compulsive increases in substance intake, and intense cravings. Such patterns contribute to the progressive disruption of personal and social functioning.^1^

SUD has detrimental effects on various bodily systems, including the immune, digestive, and nervous systems. In recent years, increasing attention has been focused on the gut-brain axis (GBA), which explores the bidirectional communication between the gastrointestinal (GI) tract and the brain, particularly regarding the reward circuits involved in SUD. Research indicates that many addictive substances share a common reward pathway, known as the dopamine system. This pathway involves dopamine neurons in the ventral tegmental area (VTA) and their projections to target regions in the limbic forebrain, particularly the nucleus accumbens (NAc).^2^ While activation of the reward system through this pathway induces pleasure or euphoria across various substances, each psychoactive substance exerts its effects through distinct pharmacological mechanisms.

The human gut microbiota is a complex ecosystem composed of bacteria, archaea, viruses, fungi, protozoa, and yeasts,^3^ which play a vital role in maintaining the integrity of the epithelial barrier, intestinal metabolism, and immune homeostasis. It is well-established that the microbiota contributes to various physiological processes, including pathogen defense,^4^ immune system modulation,^5^ fermentation of indigestible dietary fibers,^6^ and the production of bioactive peptides and proteins that regulate host energy metabolism.^7−9^ Disruption of intestinal homeostasis can profoundly impact both the gut and brain, as the GBA interacts through several biological systems, including immune, endocrine, neural, and metabolic signaling pathways, influencing overall health and disease.

Accumulating evidence in recent years indicates that addictive substances and the gut microbiota engage in a bidirectional interaction network through GBA, driving the pathological process of SUD. On one hand, addictive substances such as alcohol, tobacco, and opioids can directly or indirectly disrupt gut microbial homeostasis, leading to dysbiosis characterized by reduced beneficial bacteria and increased pathobionts.^10^ This compromises intestinal barrier function, resulting in leaky gut and translocation of bacterial endotoxins into the bloodstream, which triggers systemic and neuroinflammation. On the other hand, the dysregulated microbiota in turn modulates brain function via the GBA.^11^ Microbial metabolites, such as short-chain fatty acids (SCFAs) and tryptophan derivatives, regulate the synthesis and signaling of neurotransmitters, including GABA, serotonin (5-HT), and dopamine, thereby affecting reward and emotional circuits.^12^ Metabolites like SCFAs also exert epigenetic effects, such as inhibiting histone deacetylases (HDAC) or modulating DNA methylation, thereby altering the expression of addiction-related genes (*e.g.*, dopamine receptors and µ-opioid receptors) and persistently reshaping reward circuit plasticity.^13−15^ Additionally, gut bacteria can produce neurotransmitters and interact with the vagus nerve (VN) and immune signaling pathways, collectively modulating hypothalamic-pituitary-adrenal (HPA) axis activity and reward circuit function, ultimately promoting drug craving and relapse.^16^^,^^17^

Currently, there is no universally effective treatment for SUD, therefore, elucidating the role of the GBA in SUD is crucial for developing targeted therapeutic strategies. This review provides an overview of recent research progress on the involvement of gut microbiota and the most common substances, including alcohol, cocaine, tobacco, opioids, and methamphetamine. It further explores the potential pathogenesis underlying the relationship and intrinsic connections linking gut microbiota and SUD. By analyzing the complex and diverse interactions, we aim to reveal the potential regulatory role of microbiota within addiction pathways and emphasize its importance as a future therapeutic target.

## Alcohol

Alcohol is one of the most abused substances, affecting multiple pathways within the central nervous system (CNS). Alcohol causes physical dependence by interacting with Gamma-Aminobutyric Acidergic (GABAergic) synapses, glutamatergic signaling, and dopamine release in the midbrain’s limbic system.^18^ In addition to directly interacting with neurotransmitters, alcohol is associated with significant gut damage, characterized by disruptions in the gut microbiota, alterations of intestinal metabolism, elevated intestinal permeability, impaired nutrient absorption, and altered function of mucosal immune cells.^19^ Alcohol addiction significantly alters the composition and function of the gut microbiota, leading to intestinal dysbiosis.^1^^,^^20^

The reduction of SCFAs-related bacteria is most pronounced in alcohol use disorder (AUD) individuals, such as *Akkermansia, Alistipes, Bacteroides, Clostridium, Faecalibacterium, Ruminococcus,* and *Parabacteroides.*^20−26^ SCFAs primarily consist of acetate, propionate, and butyrate. The phylum *Bacteroidetes* mainly produces acetate and propionate, whereas the phylum *Firmicutes* is primarily responsible for butyrate production.^23^ SCFAs play an important role in maintaining intestinal barrier function, regulating immune responses, and influencing host metabolism.^27^ Butyrate alleviates intestinal inflammation and promotes homeostasis by activating GPR109a, which enhances the tolerogenic responses of colonic macrophages and dendritic cells.^27^^,^^28^ SCFAs can drive the differentiation of naïve T cells into regulatory T cells, which secrete Interleukin (IL)-10 and help maintain intestinal immune homeostasis, preventing excessive inflammatory responses.^29^ Moreover, SCFAs contribute to the maintenance of intestinal barrier function by promoting the expression of tight junction proteins, thus enhancing epithelial barrier integrity and preventing the translocation of harmful substances and pathogens into the bloodstream.^30^^,^^31^ SCFAs also modulate the gut microbiota by creating a favorable anaerobic environment that supports the growth of beneficial bacteria while inhibiting the proliferation of potential pathogenic microbes.^32^ Additionally, alterations in mucosa-associated microbiota may compromise intestinal function in individuals with AUD. The mucus layer acts as a physical barrier that prevents harmful microorganisms and their products from coming into contact with the epithelial cell surface.^33^ *In vitro* studies have shown that SCFAs regulate mucus layer thickness and participate in the repair process during mucosal damage.^34^^,^^35^ SCFAs can regulate the release of hormones such as glucagon-like peptide−1 (GLP−1) and peptide YY (PYY) from intestinal endocrine cells via the VN, indirectly influencing the brain's reward system and stress response.^36^^,^^37^

Different microbial species exhibit varying degrees of tolerance to bile acids. Compared to Gram-positive bacteria, Gram-negative bacteria generally demonstrate greater resistance to bile.^38^ *Salmonella* species show notable bile tolerance, for example, ox bile has minimum inhibitory concentrations of 18% for *Salmonella typhi* and 12% for *Salmonella typhimurium*, with minimum bactericidal concentrations of 18% and >60%, respectively. *Escherichia coli* also exhibits strong bile resistance and can grow even in high-concentration bile environments.^38^ *Lactobacillus* and *Bifidobacterium* can better survive in the gut by producing bile salt hydrolases that reduce the toxicity of bile acids.^39^ In contrast, *Helicobacter pylori* is bile-sensitive, which may explain why it has never been isolated from fecal or gallbladder specimens.^40^^,^^41^ Alcohol significantly affects bile acid synthesis, conjugation, and microbial transformation by directly regulating host gene expression (*e.g.*, CYP7A1) and indirectly altering gut microbiota composition related to bile-tolerant.^20^^,^^42^^,^^43^ These changes lead to bile acid metabolism disorders, impaired intestinal barrier function, and systemic inflammation.^44^ Individuals with AUD display a reduced abundance of *Bacteroides, Clostridium,* and *Ruminococcus*^45^^,^^46^ related to bile salt metabolism*.* Bile acids metabolites (*e.g.,* taurine and glycine) can serve as nutrient sources for microorganisms, influencing the growth and metabolism of the gut microbiota.^47^^,^^48^ Bile acids are also ligands for the pregnane X receptor (PXR), which plays a key role in maintaining intestinal homeostasis. Downregulation of PXR increases intestinal permeability, reduces tight junction protein expression, and elevates levels of pro-inflammatory cytokines such as Tumor Necrosis Factor-ɑ (TNF-*α*) and IL−1.^49^^,^^50^ In addition, bile acids can activate G protein-coupled receptors (GPCRs) such as G protein bile acid receptor (TGR5), promoting an anti-inflammatory shift in cellular phenotype, enhancing IL−10 production, and influencing intestinal endocrine function and inflammatory responses.^50^^,^^51^ More importantly, the gut microbiota can indirectly modulate farnesoid X receptor signaling by altering the composition and levels of bile acids, thereby participating in the regulation of neuroinflammation and CNS function.^51−53^

Alcohol consumption directly damages the tight junctions of intestinal epithelial cells, as evidenced by the dysregulated expression and/or altered localization of key tight junction proteins, particularly Zonula occludens−1 (ZO−1) and Claudin−1, in intestinal epithelial cell lines exposed to ethanol or acetaldehyde. This disruption ultimately leads to increased intestinal permeability (IP).^54^ Ethanol-induced activation of inducible nitric oxide synthase (iNOS) leads to microRNA−212 overexpression and reduced ZO−1 levels in Caco−2 cells and mice, ultimately resulting in increased IP.^55^^,^^56^ Acute ethanol exposure has been found to reduce IL−22 levels in the intestine, suppressing the expression of antimicrobial peptides (AMPs), Regenerating family 3β (Reg3β) and Reg3γ, thereby increasing IP.^57^ IP is closely associated with the gut microbiota.^58^ Alcohol-induced increase in IP can activate the VN, and subsequently affects brain regions associated with emotion, cognition, and behavioral regulation, including the thalamus, hippocampus, amygdala, and prefrontal cortex. As a central pathway of the GBA, the VN transmits signals related to gut inflammation and oxidative stress to the nucleus tractus solitarius (NTS) in the CNS.^59^ Alcohol increases IP, allowing bacterial products such as lipopolysaccharide (LPS) and peptidoglycan (PGN) to translocate from the gut into the bloodstream.^60^^,^^61^ This triggers the release of pro-inflammatory cytokines, such as TNF-*α* and IL−6, which activate microglia either by signaling through the VN or by directly crossing the blood-brain barrier (BBB), leading to neuroinflammation.^62^ Neuroinflammation further damages the BBB, creating a vicious cycle that exacerbates neuronal dysfunction and neurotransmitter imbalance, such as reduced dopamine and brain-derived neurotrophic factor (BDNF), manifesting as anxiety, cognitive impairment, and alcohol craving.^63^^,^^64^ Furthermore, elevated IL−8 levels are positively correlated with heightened IP,^64^ whereas IL−10 levels are inversely associated with escalated alcohol intake, likely through modulation of the Gamma-Aminobutyric Acid (GABA) signaling in the amygdala.^65^ These findings collectively suggest a pathway whereby alcohol-induced gut dysfunction and inflammation may influence neurobehavioral mechanisms of addiction, emphasizing the GBA as a potential therapeutic target.

Gut dysbiosis related to alcohol abuse has been linked to reduced levels of reward-related neurotransmitters such as 5-HT and dopamine. Microbial and metabolic changes, especially under compulsive alcohol-seeking conditions, have been associated with alterations in striatal dopamine receptor expression, potentially driving addiction behaviors.^66^ The gut microbiota plays a crucial role in the synthesis and metabolism of certain neurotransmitters and their precursors.^67−69^ In individuals with AUD, the abundance of gut microbes such as *Akkermansia*, *Alistipes*, *Bacteroides*, and *Clostridium* is reduced.^54^^,^^69^ Consequently, this leads to a shift in Tryptophan (Trp) metabolism toward the kynurenine pathway, decreasing 5-HT production, which affects mood and reward circuits, thereby exacerbating depressive and anxious behaviors in individuals with alcohol dependence.^67^

**Figure 1.**
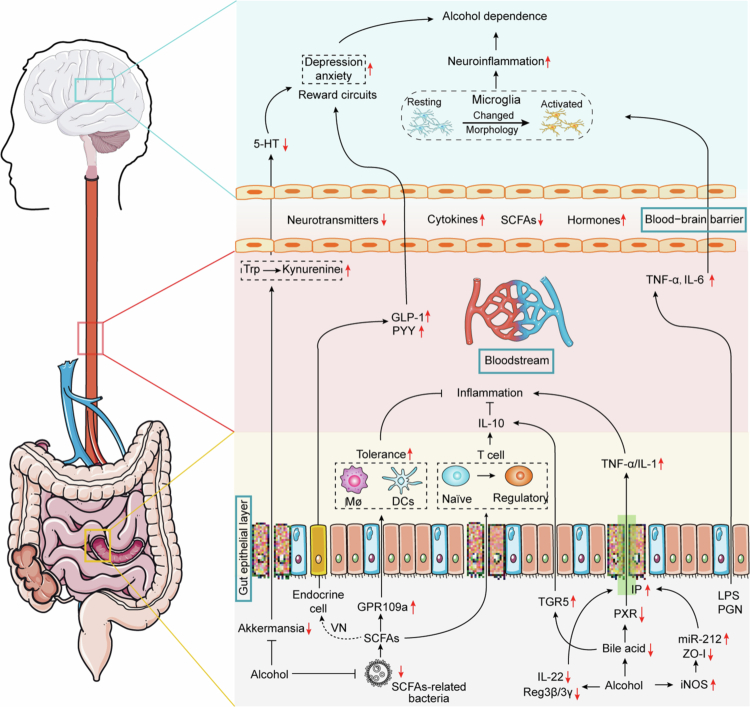
Mechanisms of gut microbiota on alcohol addiction. Alcohol reduces bile acid synthesis and PXR expression, thereby increasing intestinal barrier permeability. The heightened intestinal permeability allows more bacterial metabolites such as SCFAs, LPS, and PGN to enter the bloodstream. After crossing the blood-brain barrier, these metabolites promote neuroinflammation by activating microglia, ultimately contributing to alcohol dependence. Additionally, alcohol directly decreases the abundance of *Akkermansia* and *Alistipes*, shifting tryptophan metabolism toward kynurenine and reducing 5-HT levels, which exacerbates alcohol-related anxiety and depressive symptoms.

The intervention strategies targeting the GBA in the treatment of alcohol addiction are receiving increasing attention. For example, supplementing chronic ethanol-fed mice with *Lactobacillus rhamnosus* GG (LGG) significantly increases the mRNA levels of intestinal tight junction proteins (ZO−1, Claudin−1) and their adaptor proteins, thereby promoting intestinal barrier integrity.^70^ LGG effectively reduces alcohol-induced increases in IP and significantly alleviates alcohol-triggered oxidative stress and inflammation in intestinal tissues of male Sprague-Dawley rats.^71^ Prebiotics play an important role in the treatment of alcohol addiction and related diseases. In mouse models, a high-fiber diet reduces alcohol-induced IP by increasing SCFA-producing bacteria, such as *Bacteroides.*^58^ Numerous studies have explored the potential of fecal microbiota transplantation (FMT) from healthy donors as a therapeutic approach for AUD. Research has shown that transferring gut microbiota from individuals with alcohol dependence into germ-free mice leads to increased alcohol preference and anxiety-like behaviors in the mice, whereas transplantation of microbiota from healthy donors reduces alcohol consumption and alleviates depressive-like behaviors.^72^ In a phase I double-blind clinical trial involving patients with alcohol-related cirrhosis, FMT is a safe and effective intervention for reducing alcohol cravings and decreasing alcohol intake in the short term.^73^ Recent research suggests that gut microbiota-derived bacterial extracellular vesicles (bEVs) hold promise as a novel therapeutic target for alcohol addiction. Studies found that bEVs isolated from the gut microbiota of rats with high alcohol intake, when administered intraperitoneally or orally to alcohol-avoidant Wistar rats, increased their voluntary alcohol consumption by up to 10-fold (*p* < 0.0001). This effect is independent of bacterial colonization, indicating that bEVs alone are sufficient to directly induce behavioral changes, and do not cause systemic or brain inflammation. Crucially, bilateral vagotomy completely blocked the bEV-induced increase in alcohol intake, confirming the VN as a key signaling pathway.^74^ Chronic ethanol exposure (CEE) disrupts the gut microbiota in mice and induces anxiety-like behavior alongside hippocampal neuroinflammation; however, probiotic intervention effectively alleviates these symptoms. Further research revealed that bEVs are key carriers within the microbiota-gut-brain axis: bEVs from CEE mice enter the brain and trigger neuroinflammation and anxiety-like behavior, whereas probiotic-modulated bEVs significantly mitigate ethanol-induced neuroinflammation and anxiety-like phenotypes. This dual regulatory role likely stems from the ability of these vesicles to modulate inflammatory pathway activity and neurotransmitter levels within the hippocampus.^75^ The mechanisms between GBA and alcohol are illustrated in Figure 1.

In summary, targeting the gut microbiota through interventions such as probiotics, prebiotics, FMT and bEVs holds promising potential for alleviating alcohol addiction and its associated physical and psychological complications. However, further research is needed to elucidate the underlying mechanisms and validate the long-term efficacy and safety of these approaches in clinical settings.

## Cocaine

Cocaine is a potent neuroactive psychostimulant that induces neural activation, resulting in heightened sympathetic and behavioral responses and significant addictive potential cocaine’s primary action on the limbic system, particularly in areas dense with dopamine-responsive cells, produces its characteristic effects of intense euphoria and reward, leading to compulsive drug-seeking behavior and addiction.^76−78^

Cocaine use has been associated with GI symptoms such as anorexia, nausea, vomiting, diarrhea, and reduced GI blood flow, leading to gut microbiota imbalance in both *in vivo* and *in vitro* studies.^79^ Cocaine users exhibit alterations in gut microbiota composition, with an increased abundance of Bacteroidetes and decreased *Firmicutes.*^80^ Exposure to cocaine in mouse models induces significant alterations in the gut microbiota, characterized by the specific depletion of core SCFA-producing bacterial taxa/genera/families such as *Mucispirillum, Ruminococcaceae, Pseudoflavonifractor, Butyricicoccus,* and *Lachnospiraceae.*^81^ Butyrate serves as the primary energy source for colonic epithelial cells; its deficiency leads to impaired colonic epithelial cell function. Moreover, the lack of SCFAs may impair the regulation of histone acetylation, thereby regulating the expression of addiction-related genes.^82−84^ In male Sprague-Dawley rats subjected to gut microbiota depletion, both significantly enhanced motivation for low-dose cocaine and increased cue-induced drug-seeking behavior following prolonged abstinence were observed, concomitantly accompanied by altered transcriptional profiles in the NAc; crucially, supplementation with bacterially-derived SCFAs metabolites completely reversed these behavioral alterations induced by microbiota depletion, as well as the changes in NAc transcription.^85^ This suggests that the SCFA-epigenetic regulatory axis plays a modulatory role in the mechanisms underlying cocaine addiction.

Cocaine exposure induces intestinal microbiota dysbiosis, which is closely associated with the upregulation of proinflammatory mediators and cytokines. Cocaine modulates multiple cytokines, chemokines, and Toll-like receptor (TLR) signaling pathways, particularly activating TLR−2 and increasing levels of proinflammatory cytokines such as Monocyte Chemoattractant Protein−1 (MCP−1) and Eotaxin−1 (CCL−11), thereby fostering an inflammatory environment.^86^ This inflammatory state is further reinforced by elevated levels of Nuclear Factor kappa B (NF-κB) and IL-1β, both of which play critical roles in gut permeability, epithelial dysfunction, and neuroimmune activation. Increased IP allows translocation of LPS, which further stimulates systemic and CNS inflammation.^81^ Interestingly, cocaine self-administration behavior in rats is amplified following the intraperitoneal injection of the anti-inflammatory granulocyte colony-stimulating factor (G-CSF). Similarly, cocaine-induced locomotor activity in mice is similarly heightened.^87^

Alterations in the gut microbiota significantly impact cocaine-induced behavioral responses, which exhibit dose-dependent characteristics while remaining independent of systemic cocaine metabolism. Studies show no significant difference in benzoylecgonine levels—the primary cocaine metabolite—between cocaine-treated and antibiotic-treated mice. However, at a low cocaine dose (5 mg/kg), antibiotic-treated animals exhibit a robust place preference that is four times greater than controls, indicating heightened sensitivity. Both control and antibiotic-treated mice develop locomotor sensitization after five days of high-dose cocaine (10 mg/kg). Interestingly, oral supplementation of bacterial fermentation products, such as SCFAs (acetate, propionate, and butyrate), reverses antibiotic-induced behavioral phenotypes, suggesting that the gut microbiota influences cocaine-mediated behaviors potentially via glutamatergic and dopaminergic systems.^88^ Colonization with the probiotic *L. rhamnosus* effectively mitigated cocaine-induced intestinal oxidative stress and inflammation, concurrently suppressing glial cell activation in the CNS alongside a reduction in cocaine-associated locomotor activity.^81^ These findings confirm that the gut microbiota regulates cocaine’s behavioral effects. Recent studies have further elucidated the bidirectional modulatory effects of the gut microbiota on cocaine-induced neurobehavioral responses. Intervention studies substantiate that administering L. rhamnosus suppressed cocaine-associated gut inflammation, CNS glial activation, and hyperlocomotion.^81^ Cocaine exposure elevates intestinal norepinephrine, which activates the QseC receptor, a histidine kinase receptor, in γ-Proteobacteria, such as Citrobacter rodentium and E. coli HS, thereby promoting their colonization by these bacteria. These colonizing bacteria consume glycine as a nitrogen source, leading to significant depletion of glycine within the intestinal lumen and cerebrospinal fluid. This systemic reduction in glycine levels modulates neuroplasticity in the nucleus accumbens, ultimately potentiating cocaine-induced behavioral responses. Critically, these behavioral enhancements are reversed by either exogenous glycine supplementation or genetic ablation of the bacterial glycine transporter (cycA), establishing glycine metabolism as a mechanistically central regulator of microbiota-dependent modulation of cocaine-associated addictive behaviors.^89^ Cannabidiol (CBD) intervention significantly reduces cocaine withdrawal-induced conditioned place preference (CPP) while concomitantly restoring gut microbiota β-diversity to baseline levels in mouse models. CBD specifically expands anti-inflammatory bacterial populations (*e.g.*, *Firmicutes phylum*), attenuating the neural consolidation processes of reward memory via the gut microbiota-immune axis. CBD effectively disrupts the core pathological cycle of cocaine addiction through synergistic modulation of intestinal microecological homeostasis and neuroplasticity pathways.^90^ Notably, gut-targeted therapies (*e.g.*, probiotics, pathogen manipulation, metabolite supplementation) not only modulate addiction-relevant behaviors but also concurrently improve key biomarkers, including neuroinflammatory markers (cytokines), neurotransmitter pathway activity (striatal dopamine/glutamate signaling), and microbial metabolite profiles (SCFAs/GABA).^81^^,^^91^ Human studies corroborate that cocaine use leads to reduced gut microbial diversity and diminished beneficial metabolites, while interventions like repetitive transcranial magnetic stimulation (rTMS) demonstrate potential to partially restore microbial balance.^92^ In summary, intestinal intervention strategies, such as probiotics, prebiotics, microbiota transplantation, or glycine/sarcosine supplementation, hold promise as novel therapeutic avenues for cocaine addiction, likely acting through multi-target regulatory mechanisms.

## Nicotine

Nicotine (Tobacco), predominantly consumed via combustible tobacco products and electronic cigarettes (e-cigs), is the most widely used addictive substance globally. It exerts potent neuropharmacological effects on the CNS and exhibits a high potential for dependence. Exposure to nicotine significantly influences the microbiota of the oral, GI, and respiratory systems.^93−96^ E-cigs, often perceived as a safer alternative to traditional cigarettes, pose non-negligible risks to microbiota.

Both e-cigs and conventional cigarettes alter the oral microbiome.^97^ Tobacco smoke can alter the pH and oxygen tension in the oral cavity, inducing a localized anaerobic environment that promotes pathogenic microbial colonization and drives the establishment of a pro-inflammatory microenvironment.^98^^,^^99^ Under these conditions, smokers exhibit significant enrichment of pathogenic genera such as *Veillonella*, *Leptotrichia*, and *Prevotella*, while protective taxa, including *Haemophilus* and *Neisseria* are markedly reduced.^100^ A study investigating the oral microbiota of tobacco smokers in the Middle East further validated the aforementioned microbial alterations and revealed associations between nicotine dependence levels and specific microbial abundances.^101^ The study found that the relative abundances of *Streptobacillus hongkongensis*, *Fusobacterium massiliense*, and *Prevotella bivia* were significantly elevated in the oral microbiota of individuals with high nicotine dependence. Compared to never-smokers, the oral microbiota of smokers exhibited significant enrichment in the tricarboxylic acid (TCA) metabolic pathway. Notably, TCA such as citric acid in the TCA cycle are potent chelators of magnesium ions, and their excessive accumulation may exacerbate host magnesium deficiency. Magnesium plays a key role in nicotine addiction by inhibiting dopamine secretion, glutamate-mediated *N*-methyl-D-aspartate (NMDA) receptor activation, and the synthesis of substance P and nitric oxide.^102^^,^^103^ The high nicotine dependence group also showed significant enrichment in xanthosine utilization; xanthosine, a catabolic product of purine nucleotides, is involved in metabolic pathways related to caffeine biosynthesis. This mechanism may explain the increased demand for caffeine intake among smokers, reflecting a nicotine-caffeine synergistic addiction.^104^

Similarly, e-cigs use disrupts the structure of the oral microbiome. Studies have shown that e-cigs increase the abundance of opportunistic pathogens such as *Candida albicans* and *Porphyromonas gingivalis*, and promote the proliferation of pathogenic bacteria, including *Streptococcus mutans*, *P. gingivalis*, and *Fusobacterium.*^105^ E-cigs aerosols contain high levels of reactive aldehydes and carbonyl compounds, which can induce oxidative stress, damage host cells, and disturb microbial homeostasis.^105^ Moreover, e-cigs use reduces levels of antimicrobial proteins in saliva—such as lysozyme and lactoferrin—thereby weakening the natural immune defense of the oral cavity.^106^ Notably, *P. gingivalis* and *Fusobacterium nucleatum* play critical roles in the development and progression of periodontal disease. These pathogens can upregulate multiple pro-inflammatory cytokines, including TNF-*α*, interferon-*γ* (IFN-*γ*), IL-1β, IL−6, and IL−8, thereby activating host inflammatory responses.^107^^,^^108^ This chronic inflammatory state is not confined to the oral cavity^100^; it may also affect the CNS via systemic circulation, triggering neuroinflammation or directly modulating the brain's reward pathways, thereby increasing sensitivity to addictive substances and promoting the development and progression of addictive behaviors.^109^ Compared to never-smokers, traditional smokers exhibit broad reductions in microbial diversity across oral and pulmonary sites (152 differentially abundant taxa), whereas e-cigs users show alterations primarily confined to the oral cavity (17 differential taxa compared to smokers), with no significant shifts in the lung microbiome and only 21 overlapping taxa affected in both oral and pulmonary compartments.^110^ The use of e-cigs is significantly associated with oral microbial dysbiosis and increased biofilm accumulation. Nevertheless, although its harm is lower than that of conventional tobacco, it still poses a substantial threat to health.^110^

Compelling evidence indicates that specific components of tobacco smoke induce gut microbiota dysbiosis. Carbon monoxide (CO) alters the gut microbiome by promoting the proliferation of bacterial species that express iron-uptake-related molecules.^111^ Furthermore, heavy metals (*e.g.*, cadmium, arsenic, chromium, iron, mercury, nickel) present in cigarette smoke may, upon ingestion, cause gut microbial dysbiosis and impair intestinal epithelial cell transport function, oxidative stress status, and inflammatory responses.^112^^,^^113^ Cigarette smoke also contains highly toxic volatile organic compounds (VOCs), such as benzene, which experimental studies demonstrate can shift the overall structural architecture of the gut microbiota.^114^ A large population-based cohort study was conducted, encompassing 288 never-smokers, 267 former smokers, and 203 current smokers,^115^ and found that the three groups exhibit no significant differences in alpha diversity, whereas beta diversity reveals significant intergroup variation, indicating that smoking status reshapes the overall microbial community structure. Compared to never-smokers, current smokers exhibit elevated relative abundance of *Actinobacteria* and reduced levels of *Firmicutes* and *Proteobacteria*. Former smokers show higher relative abundance of *Bacteroidetes* and *Tenericutes* compared to current smokers, alongside decreased *Verrucomicrobia*. Notably, no significant differences are observed between never-smokers and former smokers in these phylum-level compositional changes, suggesting that long-term smoking cessation (≥6 y) can restore the microbiota to a non-smoker state. Fan *et al.* conducted a two-sample Mendelian randomization analysis and found that smoking reduces the abundance of *Peptococcus*, *Actinobacteria*, and *Bifidobacterium*. Notably, reduced *Actinobacteria* abundance correlates with increased daily cigarette consumption, while decreased *Bifidobacterium* abundance is associated with earlier smoking initiation age.^116^ *Peptococcus* participates in Trp and tyrosine metabolism, pathways closely related to neurotransmitter precursor synthesis, thereby contributing to nicotine addiction.^117^ Actinobacteria modulate the 5-HT pathway and kynurenine pathway through Trp metabolism, playing crucial regulatory roles in nicotine addiction.^116^ *Bifidobacterium* influences smoking behavior through multiple mechanisms. First, it transmits signals to the brain via the VN to regulate dopamine levels, counteracting smoking-induced euphoria or withdrawal distress.^59^ Second, it activates the Chromogranin A/Adrenergic Receptor α2A (CGA/ADRα2A) cascade and modulates the Trp Hydroxylase-Opioid Receptor (TRP/TPH-OR) pathway to promote 5-HT biosynthesis in colonic enterochromaffin cells.^118^^,^^119^ Third, it affects neurotransmitters, including GABA and norepinephrine.^120^^,^^121^ Furthermore, *Bifidobacterium* metabolites and components exhibit close associations with the CNS: SCFAs influence microglial morphology and function,^122^ acetate demonstrates therapeutic potential for preventing cognitive impairment,^123^ and PGN can penetrate the blood-brain barrier to transmit information.^124^ Overall, alterations in metabolites resulting from gut microbiota dysbiosis affect the central nervous system, potentially eliciting fear or anxiety-like emotions and inducing depression, thereby increasing the risk of smoking initiation or cessation failure.

Nicotine exposure (conventional cigarettes and e-cigs) induces microbiota dysbiosis, exhibiting sex-specific differences. Male nasal microbiota exhibits significantly higher *α*-diversity than females, with enrichment of *Abiotrophia* and *Finegoldia* genera, while females are characterized by *Yaniella* enrichment. Smokers and e-cigs users show elevated IL−8 expression in nasal lavage fluid, accompanied by relatively reduced levels of lactoferrin and IgA, suggesting that microbiota dysbiosis may drive inflammatory responses.^125^ Mouse experiments have demonstrated that sex modulates nicotine's effects on gut microbiota. Nicotine reduces *α*-diversity only in female mice, with no changes in males. It alters community structure in both sexes and drives convergence between them. *Odoribacter* significantly decreases in females, while the *Firmicutes*/*Bacteroidetes* ratio significantly decreases in males but shows no change in females. Chi *et al.* directly demonstrated the sex-specific effects of nicotine exposure on gut microbiota and gut-brain axis metabolic signaling in male and female mice.^126^ Nicotine-exposed male mice exhibit increased abundance of *Turicibacteraceae*, while female mice show significant reductions in *Christensenellaceae* and *Anaeroplasmataceae*. Female mice demonstrate comprehensive elevations in neurotransmitters, including glutamate, GABA, glycine, and serine, potentially enhancing nicotine's rewarding effects. Male mice show significant decreases in glycine, serine, and aspartate, potentially attenuating NMDA receptor activation and reducing nicotine dependence.^127−129^ Nicotine-induced metabolite changes may contribute to sex differences in nicotine addiction through alterations in the balance of neurotransmitter pools, consistent with clinical observations of higher smoking cessation difficulty in women and greater nicotine intake in men.^126^^,^^130^

Currently, therapeutic strategies targeting gut microbiota for nicotine addiction remain underdeveloped. Probiotics, such as the aforementioned *Bifidobacterium*, can improve nicotine dependence by regulating SCFAs and neurotransmitters. Additionally, *Bacteroides xylanisolvens* can degrade nicotine in the gut, reducing nicotine accumulation in the intestine and delaying the progression of non-alcoholic fatty liver disease (NAFLD) in smokers and animal models.^131^ These findings suggest that gut microbiota can serve as an auxiliary therapeutic strategy for nicotine addiction, potentially alleviating nicotine dependence and associated metabolic and inflammatory disorders.

## Opioids

Opioids which include compounds that interact with mu, delta, and kappa opioid receptors on nerve cells, have extensive effects throughout the body.^132^ Their initial interactions often occur in the GI tract.^133^ Opioid use, including agents like tramadol, methadone, and morphine, markedly disrupts gut microbiota composition, leading to reduced bacterial diversity and shifts in microbial abundance. The analgesic tramadol demonstrates dose- and time-dependent bactericidal activity against *E.coli*, *Staphylococcus epidermidis*, *Staphylococcus aureus*, and *Pseudomonas aeruginosa in vitro,*^134^ while subcutaneous injections of tramadol in mice inhibited *S. aureus* growth but not *P. aeruginosa.*^135^ Similarly, methadone shows antimicrobial effects against *S. aureus*, *P. aeruginosa*, and *Streptococcus marcescens in vitro.*^136^ In contrast, morphine significantly alters gut microbiota composition after short-term exposure.^137^ In murine studies, chronic morphine exposure was associated with the selective depletion of beneficial genera such as *Bifidobacteria* and *Lactobacillus*, contributing to microbial dysbiosis.^138−140^ In addition, a decreased abundance of beneficial gut bacteria *Bacteroidaceae* and *Ruminococcaceae* was observed in tramadol-, methadone-, and morphine-exposed patients,^141^ while heroin or prescription opioid users exhibited reduced microbiota diversity, with lower levels of *Roseburia* and *Bilophila.*^142^ These shifts in gut microbiota composition due to opioids correlated with alterations in intestinal motility, permeability, and inflammation.^143^ These changes may further interact with the brain's reward circuits, influencing opioid-related behaviors and withdrawal symptoms.^144^Germ-free mice did not exhibit typical withdrawal symptoms, while mice with TLR2 gene knockout demonstrated a reduced withdrawal response, implicating that targeting the TLR2-mediated gut microbiota could provide a promising approach to alleviate withdrawal symptoms and improve treatment outcomes for morphine use disorder.^145^ Furthermore, morphine withdrawal induces sex-specific alterations in gut microbiota composition and metabolic profiles. Following morphine withdrawal, female mice exhibit a significant increase in the abundance of *Proteobacteria*, accompanied by disrupted Trp metabolism. This dysregulation manifests as activation of the kynurenine pathway and accumulation of neurotoxic metabolites, thereby exacerbating anxiety-like behaviors. In contrast, male mice demonstrate relative enrichment of *Bacteroidota* alongside reduced abundance of *Actinobacteria* and *Firmicutes*. These microbial shifts primarily correlate with diminished locomotor activity, while anxiety-like behaviors remain unaltered. This behavioral phenotype is closely associated with inflammatory response activation.^146^ These findings underscore the complex relationship between opioids and gut microbiota, highlighting the gut microbiota as a potential target for mitigating opioid-related GI and neurological effects.

Opioids induce gut microbiota dysbiosis and significantly disrupt the metabolic homeostasis of SCFAs. Chronic morphine administration in mice markedly reduces fecal levels of butyrate and propionate,^147^ while individuals undergoing methadone maintenance treatment exhibit a synchronous decline in fecal acetate, propionate, and butyrate.^148^ These alterations are closely associated with structural disruption of the microbiota, characterized by a significant reduction in the abundance of SCFA-producing core taxa, including *Firmicutes* and *Akkermansia muciniphila.*^149^ Critically, antibiotic-mediated depletion of the gut microbiota exacerbates the reduction in SCFAs following morphine exposure, demonstrating that opioids directly suppress SCFA biosynthesis by perturbing microbial ecological stability.^150^ SCFAs play a pivotal role in mediating opioid-induced tolerance, addiction, and withdrawal. Opioids compromise intestinal epithelial barrier integrity by disrupting tight junctions, leading to increased paracellular permeability, as evidenced by elevated FITC-dextran flux. This barrier dysfunction permits bacterial translocation and subsequent systemic inflammation.^147^ Conversely, both butyrate and other metabolites derived from *A. muciniphila*, distinct from SCFAs, have been shown to restore epithelial barrier function, thereby preventing microbial translocation and dampening systemic inflammatory cascades. Consistent with this, methadone maintenance treatment is associated with elevated plasma levels of pro-inflammatory cytokines IL−6 and TNF-*α.*^148^ SCFAs counteract this inflammation primarily through their activity as HDAC inhibitors, which downregulate inflammatory gene expression and modulate transcriptional programs within reward-related brain regions such as the NAc, including key immediate-early genes like *Egr2* and *Egr4.*^150^ Furthermore, SCFAs can cross the blood-brain barrier to directly influence dopaminergic signaling or indirectly modulate central reward circuitry via vagal afferent pathways.^151^ Interventions targeting the microbiome or supplementing SCFAs effectively reverse opioid-induced behavioral deficits. Oral administration of butyrate prevents the development of morphine-induced antinociceptive tolerance^147^ Supplementation with a mixture of SCFAs completely restores morphine-induced CPP deficits caused by antibiotic-mediated microbiota depletion.^150^ In a fentanyl self-administration model, SCFA repletion normalizes reward pathway function and reverses the enhanced motivation and drug-seeking behavior caused by microbiota disruption.^152^ Moreover, the gut microbiota, acting via SCFAs, regulates withdrawal severity: antibiotic-induced microbiota depletion attenuates naloxone-precipitated morphine withdrawal symptoms, whereas exogenous SCFA supplementation reduces fentanyl-seeking behavior, indicating that SCFA levels are a critical determinant of withdrawal intensity and relapse vulnerability.^152^^,^^153^

Opioids exacerbate irinotecan (CPT−11)-induced GI toxicity by increasing the abundance of *β*-glucuronidase-producing bacteria and resulting in SN−38 accumulation in the small intestine.^154^ Opioid use disrupts gut microbiota homeostasis, compromises intestinal immunity and increases susceptibility to infections.^155−157^ Studies have demonstrated a marked increase in chemokine expression in the intestinal tissue of morphine-treated mice, accompanied by elevated neutrophil infiltration that contributes to gut microbiota dysbiosis.^158^ Opioids can increase IL−2, IL−4, IL−6, IL−10, TNF-*α* and IFN-*γ*. subsequently, the intestinal barrier was disrupted by activating the TLR2/4 pathway, leading to persistent bacterial transmission^159^ and leakage of microbial components such as LPS, lipoteichoic acid (LTA), and endotoxins into the systemic circulation. The latter binds to receptors on the circulating immune cells to initiate cytokine production.^160^ The cytokines not only disrupt the tight junction of gut epithelia but also into the blood circulation and deposited into the CNS^138^^,^^140^ thereby regulating the functional connectivity of brain regions related to behavioral changes such as intoxication and withdrawal.^161^ Alterations in the thalamus have been identified in rats exposed to morphine. Changes in gut microbiota diversity and bacterial abundances precede these anatomical features, and emerging neuroinflammation is a crucial link mediating communication between the gut and the thalamus.^162^ In microbiota-depleted rodents, increased recruitment of Fos^+^ neuronal ensembles was identified in the basolateral amygdala (BLA) and central nucleus of the amygdala (CeA) in an oxycodone intoxication and withdrawal model. The BLA connects with the CeA, which plays an integral role in withdrawal. Increased Fos^+^ neurons in the BLA send projections to the NAc, a central brain area underlying reward.^163^ Microglia in the NAc exhibit higher expression of TLR2 after chronic morphine treatment. This activation of microglia was markedly inhibited, accompanied by attenuated behavioral signs of morphine withdrawal in TLR2 knockout mice.^164^

The gut microbiota influences DNA methylation patterns.^165^ Choline-utilizing gut bacteria decrease DNA methylation in various brain regions by competing for choline, leading to increased anxiety in mice.^166^ Morphine alters gut microbiota related to DNA methylation.^140^^,^^166^ Morphine use has been related to a decrease in *Lactobacillus* levels in the gut,^167^ which has been shown to attenuate abdominal pain and alleviate depression and anxiety by sustaining levels of IFN-*γ*^168^ and mediating the expression of the opioid receptor gene (OPRM) in intestinal epithelial cells via NF-κB-dependent mechanism. DNA methylation of the OPRM promoter correlates with opioid use,^169^ and is influenced by a single nucleotide polymorphism (SNP) at position + 188 of the OPRM gene promoter.^170^ This SNP is significantly associated with the onset and treatment of opioid addiction.^171^ These results imply that opioid addiction leads to complex changes in DNA methylation levels, partly mediated by the microbiota. Nevertheless, the linkage between the microbiota and DNA methylation-mediated opioid addiction and withdrawal symptoms remains insufficiently understood.

Interventions such as probiotics, prebiotics, SCFA supplements, and FMT can improve opioid-induced microbial dysbiosis, reduce withdrawal symptoms, delay the development of tolerance, and alleviate comorbid psychiatric and psychological symptoms. Probiotics enriched with *Bifidobacteria* and *Lactobacillaceae* significantly modulate gut inflammation in morphine-treated mice. This is a modulation that prolongs the analgesic effects of morphine and attenuates the development of antinociceptive tolerance.^140^ Oral administration of *Lactobacillus acidophilus* NCFM induces the expression of *μ*-opioid and cannabinoid receptors in intestinal epithelial cells, thereby exerting gut analgesic effects comparable to those of morphine.^172^ Crawford *et al.* found that the ketogenic diet alleviate abnormal pain behaviors induced by chronic morphine treatment in male mice by restoring SCFA-producing bacteria.^173^ This dietary approach may serve as an adjunctive strategy for analgesic management in patients receiving long-term opioid therapy. Additionally, studies indicate that supplementing with a diet rich in n-3 polyunsaturated fatty acids (n-3 PUFAs) during opioid exposure and withdrawal significantly increases the abundance of *Bifidobacterium* and *Allobaculum*. Among these, *Bifidobacterium* enhances opioid bioavailability by degrading glucuronides in the intestinal lumen. This action thereby improves withdrawal outcomes. In addition, n-3 PUFA-induced enrichment of *Allobaculum* is closely associated with reduced anxiety-like behaviors and may exert neuroprotective effects by suppressing anxiety-promoting taxa such as *Akkermansia.*^174^ Collectively, n-3 PUFAs may alleviate opioid-induced behavioral and neurophysiological abnormalities through modulation of gut microbiota composition. Furthermore, Xie *et al.* reported that molecular hydrogen, particularly coral calcium hydride 200 (CCH 200), can alleviate depressive and anxiety-like symptoms in individuals with opioid addiction and modulate gut microbiota composition. It facilitates the extinction of morphine-associated behaviors and reduces the risk of relapse. The underlying mechanism may involve decreasing the *Firmicutes/Bacteroidetes* ratio and increasing the relative abundance of beneficial bacteria such as *Lactobacillus* and *Alloprevotella*, thereby significantly ameliorating morphine-induced gut microbiota dysbiosis.^175^ These findings suggest that the gut microbiota may serve as a key potential mechanism through which molecular hydrogen exerts its therapeutic effects in opioid addiction.

**Figure 2.**
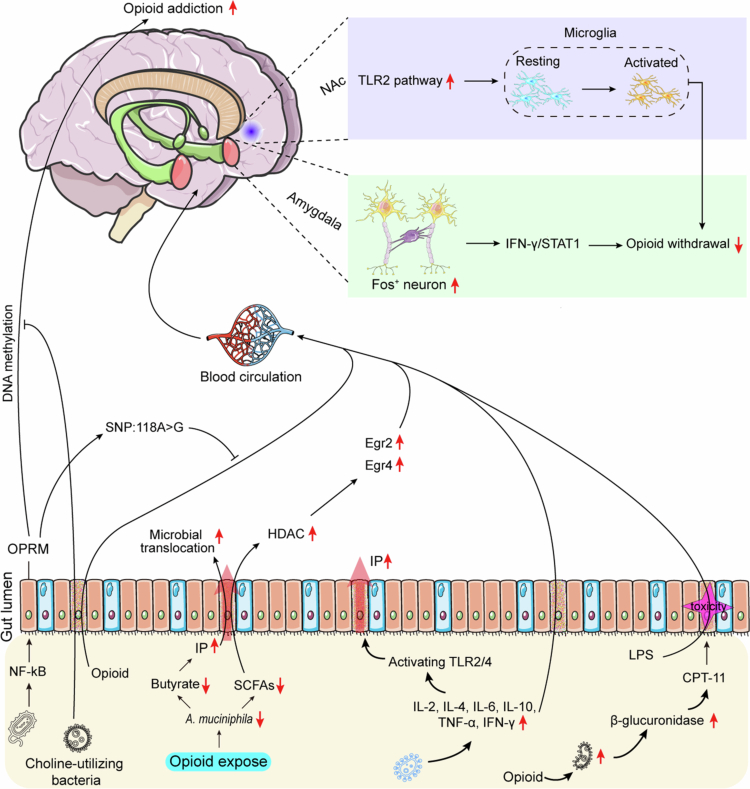
Mechanisms of gut microbiota on opioid addiction. Opioid-related bacteria can activate the TLR2/4 pathway by increasing cytokines such as IL−2, IL−4, and TNF-*α*, thereby enhancing intestinal barrier permeability. Opioids can increase the abundance of bacteria involved in *β*-glucuronidase activity, upregulating CPT−11 and exacerbating GI toxicity, further damaging the intestinal barrier function. After entering the circulatory system, the bacterial metabolite LPS activates the TLR2 signaling pathway in the NAc region and reduces opioid withdrawal symptoms by promoting microglial activation. Furthermore, FOS^+^ neurons in the amygdala regulate opioid withdrawal symptoms via the IFN/STAT1 pathway. Choline-utilizing gut bacteria alleviate opioid addiction symptoms by reducing DNA methylation levels in multiple brain regions.

FMT has emerged as a promising therapeutic strategy in the treatment of opioid addiction.^176^ FMT restores the diminished butyrate synthesis capacity in the intestines of mice that have been chronically exposed to morphine. Butyrate enhances intestinal barrier function and suppresses systemic inflammation; it also maintains opioid receptor sensitivity, thereby effectively delaying the development of morphine analgesic tolerance.^177^ Furthermore, transplantation of gut microbiota from morphine-naïve mice significantly alleviates naloxone-precipitated withdrawal symptoms. The underlying mechanisms include restoration of intestinal barrier integrity, reduction of bacterial translocation, and inhibition of TLR2/4-mediated inflammatory pathway activation.^140^ Notably, transplantation of microbiota from morphine-withdrawn mice induces anxiety-like behaviors in recipient animals; conversely, FMT ameliorates anxiety phenotypes by reshaping gut microbial balance, downregulating aberrant expression of serotonin receptors (*e.g.*, Htr6) in the amygdala, and reversing Trp metabolic dysregulation.^146^ However, current explorations of FMT as a treatment for opioid addiction are limited to preclinical studies, and variations in individual microbiota may contribute to differences in opioid tolerance and withdrawal symptoms. The mechanisms underlying the interaction between GBA and opioids are illustrated in Figure 2.

## Methamphetamine

Methamphetamine (METH) is a highly addictive neurotoxic substance with primary effects on the brainstem, where it induces addiction through mechanisms involving oxidative stress, activation of transcription factors, DNA damage, excitotoxicity, BBB impairment, microglial activation, and apoptotic pathways.^178^ METH abuse has a profound impact on the profiles and functions of gut microbiota. METH exposure alters the *α* and *β* diversity of gut microbiota in mice, increasing the relative abundance of pathogenic microbiota and reciprocally decreasing the beneficial bacterial taxa.^179^ Studies on METH-treated rats have shown a marked decrease in propionate-producing bacteria such as *Phascolarctobacterium*, along with an increase in the abundance of the *Ruminococcaceae* family, *Proteobacteria*, and *Fusobacteria.*^179^ METH exposure is associated with a slight increase in overall microbial biodiversity, with a significantly higher abundance of *Rumen cocciz* compared to control groups, and is linked to METH-induced neurotoxicity and cognitive impairments.^180^ Changes in microbiota abundance in METH-exposed mice were significantly correlated with depression-like behaviors and neurotoxicity.^181^ On the one hand, gut microbiota produces metabolites that indirectly regulate the gut-brain axis. Microbial metabolites such as Trp and SCFAs play a role in connecting to the gut-brain axis, thereby regulating neural function.^182^^,^^183^ *Anaerostipes* both produces SCFAs and facilitates Trp metabolism.^184^^,^^185^ METH withdrawal resulted in a reduction of *Anaerostipes*, thereby inhibiting the transmission of Trp microbial metabolic signaling mediated by the Aryl hydrocarbon receptor (AhR) pathway through immune response, and contributing to the development of anxiety and depression-like behaviors.^183^ A similar study showed that enhanced microbial Trp metabolism by *Lactobacillus reuteri* reduced anxiety-like behavior by upregulating IL−22 levels in the colon.^186^

Microbes release SCFAs that regulate G-protein-coupled receptors to mediate neurotransmitter release (*i.e.*, dopamine).^187^ Microbial dysbiosis contributes to METH-related anxiety and depression by reducing microbiota-derived SCFAs.^188^ Alpha synuclein can be translocated from the stomach to the brain more easily when there is a shortage of SCFAs, which can result in increased intestinal permeability.^189^^,^^190^ Supplementing SCFAs can alleviate METH-induced neurotoxicity and behavioral abnormalities through the Wnt/β-Catenin/GSK3β signaling pathway and anti-inflammatory effects, revealing the neuroprotective potential of the gut microbiota.^191^ Microbiota-derived SCFAs maintain intestinal barrier function by inhibiting macrophage infiltration and M2 macrophage phenotype switching,^192^ the latter is associated with METH-induced immune impairment.^193^ Microbiota-derived SCFAs could modulate METH-induced anxiety- and depression-like behaviors via the Sigma−1 receptor (SIGMAR1)/BDNF/tyrosine kinase B (TrkB) pathway.^188^ SIGMAR1 is expressed throughout the brain, and it shows protective effects against multiple mental disorders, including anxiety and depression.^194^ SIGMAR1 may serve as one of the key mediators in the bidirectional communication of the gut-brain axis. SIGMAR1 knockout induced dysbiosis of the gut microbiota with lower expression of neurotrophic factors.^195^ BDNF and its receptor TrkB are crucial anti-anxiety and anti-depression factors.^196^ Prebiotic and probiotic treatments restored cognitive function by reverting BDNF and *α*-synuclein levels in the hippocampus.^197^ *Lactiplantibacillus* and *Bifidobacterium* downregulated gut dysbiosis by increasing the hippocampal BDNF-positive cell population and BDNF level, leading to the alleviation of depression and cognitive impairment.^198^ Furthermore, SCFA-producing bacteria (*i.e.*, *Bacillus licheniformis*) can regulate gut microbiota composition and increase dopamine levels in the brain.^199^

On the other hand, gut microbiota can increase neuromodulators in the human brain, including dopamine produced by *Bacillus.*^200^ These neurotransmitters mediate gut-brain signals and regulate cognition and behavior by communicating with the limbic system.^201^ Dopamine is best known for its role in the reward system, where it plays a fundamental part in reward learning and prediction.^202^ Microbiota-derived dopamine in the gut can regulate the production of certain cytokines such as IL−4 and IFN-*γ*, thereby activating immune responses through natural killer cells.^203^ Furthermore, gut microbiota are major contributors to dopamine bioavailability in the enteric and central nervous systems.^204^ Metabolites produced by gut microbiota are associated with dopamine functioning through modulation of dopaminergic synaptic transmission. In rodents, *Prevotella* exerts protective properties against degeneration of tyrosine hydroxylase-containing dopaminergic neurons by secreting hydrogen sulfide.^205^ These findings indicate that gut microbes alter the level and function of striatal dopamine by regulating cytokine-mediated inflammation.

Additionally, gut microbiota can influence the cellular metabolic function of microglia. The transformation of microglial M2 to M1 and the subsequent change of proBDNF–p75^NTR^/mBDNF–TrkB signaling lead to a reduction of hippocampal neurogenesis and deteriorated spatial learning and memory in METH-exposed mice.^206^ Gut-derived bacterial LPS enter the circulating blood, activate microglia in the brain and consequently attenuate METH craving after METH withdrawal^207^; this process is associated with the activation of the TLR4/MyD88/NF-κB signaling pathway.^208^ LPS administration increases intestinal permeability, allowing endotoxins to travel towards the midbrain. This causes neuroinflammatory effects, which lead to a decrease of dopamine levels.^209^ LPS administration can influence brain activity by producing reactive oxygen species (ROS) through NADPH oxidase activity and result in microglial activation and dopaminergic neuronal loss.^210^

**Figure 3.**
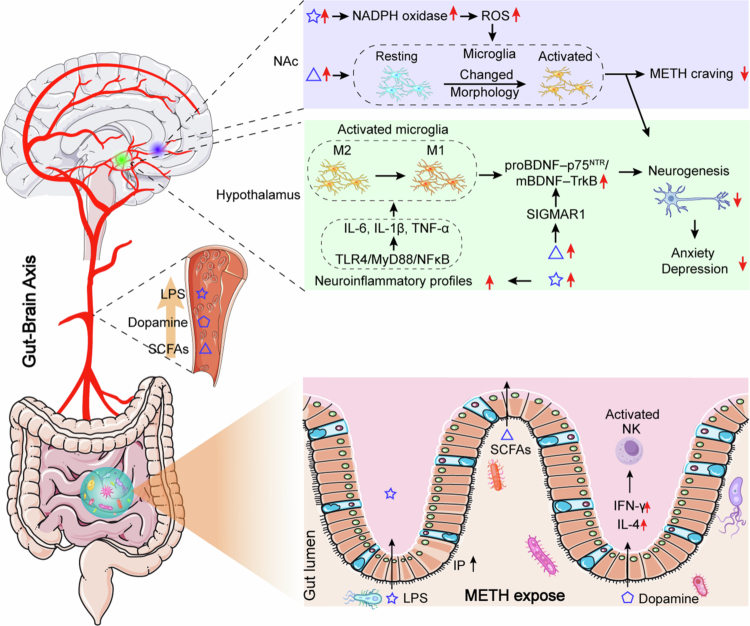
Mechanisms of gut microbiota on METH addiction. METH can increase intestinal barrier permeability, facilitating the entry of bacterial metabolites such as LPS, SCFAs and dopamine into the circulatory system. Once LPS and SCFAs reach to the NAc region, they increase ROS levels by upregulating NADPH oxidase, thereby activating microglia and reducing neurogenesis. In addition, SCFAs activate the BDNF-p75-TrkB pathway via the SIGMAR1 receptor, further suppressing neurogenesis in the hypothalamus. Similarly, LPS activates the TLR4/MyD88/NF-κB pathway and elevates IL−6, IL-1β, and TNF-α; it then promotes the shift of microglia from the M2 to the M1 phenotype. This shift further enhances the BDNF-p75-TrkB pathway in the hypothalamus, ultimately alleviating METH-induced depressive and anxiety-like behaviors.

Supplementing gut microbial metabolites, such as SCFAs, or the peroxisome proliferator-activated receptor *γ* (PPARγ) selective agonist pioglitazone, can effectively alleviate METH-induced neurotoxicity and behavioral abnormalities. SCFAs exert their effects by modulating the Wnt/β-catenin/GSK3β signaling pathway, restoring intestinal barrier function, and suppressing neuroinflammatory responses in the CNS. Moreover, both SCFAs and pioglitazone improve METH-induced neuroinflammation, anxiety-, and depression-like behaviors, as well as restore intestinal function, through VN-dependent mechanisms.^191^ METH withdrawal significantly reduces key gut microbial genera associated with TRP metabolism, including *Akkermansia*, *Bacteroides*, *Faecalibaculum*, *Desulfovibrio*, and *Anaerostipes*. This leads to a marked decrease in indole derivatives produced through the TRP metabolic pathway. A high-TRP diet effectively suppresses METH-induced depressive- and anxiety-like behaviors, and supplementation with indole−3-acetic acid (IAA), indole−3-pro-pionic acid (IPA), or indole compounds also ameliorates METH-induced behavioral abnormalities. Notably, these protective effects are significantly attenuated in AhR knockout mice, highlighting the critical regulatory role of the “microbiota-indole-AhR signaling axis” in METH-associated emotional disturbances.^183^ FMT enhances the expression of 5-HT receptor subtype 4 (5-HT4R) in the NAc and hippocampus of mice. Activation of 5-HT4R not only attenuates intestinal inflammation but also modulates dopamine release, effectively reducing METH-induced CPP behavior.^211^ Furthermore, the abundance of *Rikenellaceae* exhibits a positive correlation with anxiety and depressive symptoms in METH users, suggesting that restoring this microbial population through FMT may alleviate withdrawal-related emotional disturbances and improve mental health.^212^ Transplantation of gut microbiota from METH users into germ-free mice induces anxiety- and depression-like behaviors, whereas metformin treatment significantly ameliorates these behavioral abnormalities. Mechanistic studies indicate that metformin likely modulates gut microbiota composition, thereby affecting neuroinflammatory responses and neurotransmitter imbalances during METH withdrawal and participating in its pathophysiological processes.^212^ These findings underscore the pivotal role of gut microbiota in emotional disorders caused by METH addiction and provide a theoretical foundation for novel microbiota-targeted therapeutic strategies. The mechanisms underlying the interaction between GBA and METH are summarized in Figure 3. Tables 1 and 2 summarize the microbial effects observed in SUD and interventions targeting the GBA, respectively.

**Table 1.** Microbes’ effects in substance use disorders.

Substance	Representative microbiota shift	Key gut-to-host mechanisms	Anxiety/depression	Reward/craving	Model + dose/exposure	Citation
Alcohol	↓ SCFA/bile-salt bacteria; ↑ *Proteobacteria*	SCFA↓ → GPR109a/Treg↓; FXR/PXR/TGR5 dysregulation; LPS translocation → NF-κB, IL-1β↑	▲ Elevated anxiety-like behaviour in open-field & EPM	▲ Voluntary ethanol intake & cue-induced craving	C57BL/6J mice, Lieber-DeCarli 5% (v/v) ethanol diet, 4 weeks	[19, 50, 62, 64–67]
Cocaine	↓Butyrate taxa (*Mucispirillum*, *Lachnospiraceae*); ↑ *γ*-*Proteobacteria*, *Bacteroidetes*; ↓ *Firmicutes*	SCFA↓ → HDAC disinhibition, epigenetic reprogramming; glycine↓via *Citrobacter/E. coli* (NMDA modulation); TLR2 - NF-κB axis; LPS translocation	▲ Mild anxiety/depression (SCFA depletion, Trp dysregulation)	▲ CPP ↑ (5 mg kg⁻¹, 4×); ▲ locomotor sensitisation (10 mg kg⁻¹, 5 d); ↑cue-induced seeking; CBD blocks withdrawal-CPP	Sprague-Dawley rats; i.p. cocaine 5/10 mg kg⁻¹; ± broad-spectrum ABX; C57BL/6J ± CBD; human SUD cohorts	[73, 77, 80, 85, 89–92]
Nicotine (smoking)	Oral pathobionts ↑ (*Veillonella-Prevotella*); *Bifidobacterium*,↓ *Peptococcus*, *Actinobacteria*; ↓ *Firmicutes*/*Proteobacteria*	SCFA↓; IL−8/TNF-α↑; Mg²⁺ chelation removes NMDA blockade; Trp metabolism altered	▲ Higher trait anxiety (♀ > ♂); depressive-like phenotypes linked to Bifidobacterium loss	▲ Pack-year & plasma cotinine correlate with Actino-loss; ↑ dependence severity	0.8 mg kg⁻¹ nicotine in drinking water, 6 weeks; human smokers ≥10 cigarettes d⁻¹	[91, 102–108, 116]
Nicotine (e-cigs)	Oral *Candida*/*P. gingivalis*/*S. mutans* ↑; salivary lysozyme, lactoferrin↓	Carbonyl radicals → epithelial DNA damage; antimicrobial peptides↓; IL-1β, TNF-α↑	(Oral discomfort＞CNS)	↑ Oral biofilm; potential gateway to dependence(under study)	BALB/c mice, vanilla-flavoured e-cigs aerosol 2 × 30 min d⁻¹, 4 weeks; human cohorts	[96–101, 105]
Opioids	↓ *Bifidobacterium* & *Lactobacillus*; ↑ *β*-GUS bacteria; ↑♀*Proteobacteria*	Butyrate↓ → barrier leak↑; *β*-GUS re-activates SN−38/opioids; TLR2/4–NF-κB; kynurenine-shift (♀); DNA methylation changes (OPRM)	▲ Anxiety-like behaviour post-morphine withdrawal (♀); sex-specific differences (♂ locomotor ↓)	▲ Naloxone-precipitated somatic signs↑ tolerance, relapse risk; SCFA depletion worsens craving	Male C57BL/6: morphine 10 mg kg⁻¹ s.c., 7 d; TLR2-KO comparison; human MMT patients	[129–133, 138–147, 150–153, 165–171]
METH	↑*Ruminococcaceae/Proteobacteria/Fusobacteria;* ↓ *Anaerostipes*, SCFAs, indoles	SCFA + indole↓ → Wnt/β-catenin & AhR-IL−22 suppression; NF-κB↑; barrier permeability↑	▲ EPM & tail-suspension anxiety/depression; rescued by SCFA/Trp/indole diet, probiotics	▲ CPP & self-administration; anxiety/depression reversed by FMT, metformin, SCFAs	C57BL/6J mice, METH 1 mg kg⁻¹ i.p., 14 d; high-Trp diet 2%; FMT/metformin intervention	[162–166, 171–175, 194,195]

Model + dose reflect the most widely used paradigm in the citations; EPM: elevated-plus-maze; CPP: conditioned place preference; ABX: antibiotics; i.p.: intraperitoneal; s.c.: subcutaneous; ▲: enhanced; ↑: increased; ↓: decreased

**Table 2.** Interventions targeting the GBA.

Strategy	Substance(s)	Intervention details (form/dose/duration)	Gut-side effects (microbiota/metabolites/barrier)	CNS & behavioural effects	Evidence level + model	Citation
*Lactobacillus rhamnosus* GG	Alcohol	Live probiotic, 10⁹ CFU d⁻¹ in chow, 4 weeks	↑ ZO−1, Claudin−1 mRNA; ↓ ROS & TNF-*α* in jejunum	↓Intestinal permeability; ↓ anxiety index (open field)	Pre-clinical, male C57BL/6J, *n* = 10	[70]
High fibre/inulin diet	Alcohol	Inulin 10% kcal, 8 weeks	↑ *Bacteroides* & butyrate; mucus layer restored	↓ FITC-dextran leak; mild ↓ ethanol preference	Pre-clinical, BALB/c, *n* = 8	[58]
Healthy-donor FMT	Alcohol	50 mg stool kg⁻¹ by oral gavage, 3×/week, 2 weeks; clinical: frozen-capsule, single dose	↑ *α*-diversity, ↑ butyrate genes	↓ drinking (2-bottle choice); ↓ craving VAS (phase-I cirrhosis patients)	Mouse (GF) & human phase-I, *n* = 20	[72,73]
Probiotic-bEV vs. alcohol-bEV	Alcohol	bEV 3 µg protein i.p. daily, 14 d	Probiotic-bEV: ↓ NF-κB, IL-1β; Alcohol-bEV: opposite	Probiotic-bEV: ↓ hippocampal microglia; Alcohol-bEV: 10-fold ↑ intake	Rat (Wistar), *n* = 12	[74,75]
SCFA cocktail (acetate:propionate:butyrate 60:20:20)	Cocaine/METH	150 mM in drinking water, 21 d	Restores total faecal SCFA; ↑ HDAC1 acetylation	Normalises CPP and anxiety scores	C57BL/6J, *n* = 10	[85,88]
Glycine/sarcosine	Cocaine	1 g L⁻¹ in drinking water, 10 d	Normalises gut glycine; ↓ TLR−2 mRNA	CPP magnitude returns to baseline	Sprague-Dawley, *n* = 12	[89]
*L. rhamnosus* JB−1	Cocaine	10⁹ CFU d⁻¹ gavage, concurrent with cocaine 14 d	↓ Ileal MPO; restores Lachnospiraceae	↓ Locomotor hyperactivity; ↓ Iba−1 glial signal	C57BL/6J, *n* = 8	[81]
Cannabidiol (CBD)	Cocaine	20 mg kg⁻¹ i.p., during withdrawal 7 d	↑ Firmicutes; *β*-diversity → baseline	Blocks withdrawal-CPP reconsolidation	C57BL/6J, *n* = 9	[90]
rTMS (DLPFC, 10 Hz)	Cocaine	3 000 pulses session⁻¹ , 10 sessions	↑ Faecalibacterium, ↓ Proteobacteria	↓ Craving VAS; ↑ abstinence days	Human SUD clinic, *n* = 32	[92]
Multi-strain probiotic (Bifido/Lacto)	Nicotine; Opioids	5 × 10⁹ CFU d⁻¹ capsules, 4 weeks	↑ SCFAs; ↓ CRP & IL−6	↓ Cig/d; morphine tolerance delayed	Pilot RCT smokers (*n* = 40) & mice	[140]
*Bacteroides xylanisolvens*	Nicotine	10⁸ CFU d⁻¹ gavage, 6 weeks	Nicotine demethylated → cotinine↓; ↓ Lactate	Ameliorates NAFLD (↓ALT, ↓steatosis)	C57BL/6J + chronic smoke, *n* = 12	[131]
Magnesium supplementation	Nicotine	MgCl₂ 2 g L⁻¹ drinking water, 4 weeks	Recovers colonic Mg²⁺; ↑ Bifidobacterium	↓ Conditioned nicotine place preference	C57BL/6J, *n* = 10	[104]
Probiotic (Bifido/Lacto)	Opioids	5 × 10⁹ CFU d⁻¹ capsules, 4 weeks	↑SCFA levels; ↓gut inflammation	↓Withdrawal severity, ↓prolonged analgesia, and tolerance	Morphine-treated mice	[140]
*Lactobacillus acidophilus* NCFM	Opioids	Oral gavage, dose NR	↑μ-opioid & cannabinoid receptor expression in IECs	Gut analgesia comparable to morphine	Pre-clinical (IEC, animal)	[172]
Ketogenic diet	Opioids	90% kcal fat, 14 d pre- and post-morphine	↑ butyrate producers, ↑ βHB; ↓ IL-1β	Allodynia; ↓delayed tolerance	C57BL/6J, *n* = 10	[173]
n-3 PUFA diet	Opioids	DHA/EPA 3% kcal, 21 d	↑ *Bifidobacterium*, *Allobaculum*; ↓ *Akkermansia*	↓Anxiety-like behavior; improved withdraw outcome	BALB/c, *n* = 12	[174]
Molecular hydrogen (coral calcium hydride)	Opioids	50 mg kg⁻ ¹ *p*.o., 10 d	↓ *Firmicutes*/*Bacteroidetes ratio*; ↑ *Lactobacillus*	↓Depression-/anxiety-like behavior; ↓relapse risk	SD rats *n* = 16	[175]
Naïve-donor FMT	Opioids	100 mg stool kg⁻¹ gavage, 3× after morphine	Restores butyrate genes; ↓TLR2/4	↓Naloxone withdrawal scores; ↓ anxiety-like phenotype	C57BL/6J, *n* = 10	[140,176,177]
*β*-GUS inhibitor (uncleaved SN−38)	Opioids + CPT−11	UNC10201652 20 mg kg⁻¹ *p*.o., 3 d	Blocks bacterial *β*-GUS; ↓SN−38 stools	↓ CPT−11 diarrhoea score ; no effect on analgesia	BALB/c, *n* = 8	[154]
High-Trp/Indole diet	METH	2% tryptophan chow or 25 mg kg⁻¹ IAA gavage, 14 d	Indole-AhR-IL−22 axis restored; ↑*Akkermansia*	↓ EPM anxiety + TST immobility	C57BL/6J, *n* = 10	[183]
Pioglitazone + SCFA water	METH	Pio 20 mg kg⁻¹ *p*.o. + SCFA cocktail 21 d	↓Barrier leak, ↑ZO−1; ↓systemic IL−6	↓ Anxiety & depressive-like indices	C57BL/6J, *n* = 8	[191]
*Lactobacillus reuteri*	METH	10⁹ CFU d⁻¹, 10 d	↑ colonic IL−22;↑ indole pathway	↓ EPM anxiety	ICR mice, *n* = 12	[186]
Prebiotic/probiotic	METH	Oral; dose/duration NR	↑Hippocampal BDNF；↓α-synuclein	↓Depression；improved cognition	Pre-clinical, METH mice	[197,198]
*Bacillus licheniformis*	METH	10⁸ CFU d⁻¹, 28 d	↑ butyrate & acetate; ↑ DA precursor pool	↑ Striatal dopamine; ↑ NOR memory score	SD rats, *n* = 10	[199]
Healthy-donor FMT	METH	100 mg stool kg⁻¹ gavage, 3 d	↑ 5-HT4R mRNA in NAc & HPC; barrier repair	↓ CPP score 50%; ↓ anxiety	C57BL/6J, *n* = 10	[211]
Metformin-modulated microbiota	METH	200 mg kg⁻¹ metformin *p*.o., 10 d; donor feces to GF mice	↓ *Rikenellaceae*; ↓ IL-1β in PFC	Transfer eliminates donor-induced anxiety & depression	Germ-free C57BL/6J *n* = 8	[212]

CFU: colony-forming units; p.o.: per os (oral); i.p.: intraperitoneal; s.c.: subcutaneous; NOR: novel object recognition; GF:germ-free; βHB: *β*-hydroxybutyrate; RCT: randomised controlled trial. “Evidence level” specifies the highest tier studied: human, pre-clinical (rodent), or GF transfer. Doses/timings follow the original reports; always interpret within species-specific pharmacokinetics; ↑: increased; ↓: decrease.

## Limitations and future perspectives

Despite the significant therapeutic potential that gut microbiota offers for SUD, several critical limitations remain. Emerging evidence suggests that the gut microbiota may regulate addictive behaviors in a sex-dependent manner. However, research focusing on specific populations such as adolescents and the elderly remains insufficient, limiting the generalizability and potential for personalized applications of current findings.

Most studies rely heavily on animal models, which fail to fully replicate the complexity of human genetic diversity, environmental exposure histories, and individualized drug-use patterns. Therefore, caution must be exercised when extrapolating findings from animal experiments to human populations. Although 16S rRNA and metagenomic sequencing are widely used, the taxonomic identification, abundance quantification, and functional annotation of microbiota in these analyses are significantly influenced by multiple factors. These factors include sample purity, gene copy number variations, sequencing depth, and reference database completeness.

At the clinical level, a cross-sectional study demonstrates limited effectiveness of dynamic changes in gut microbiota in modulating addictive behaviors. Furthermore, studies’ reliance on fecal specimens limits accurate representation of mucosa-adherent microbiota composition and associated microenvironmental alterations along the GI tract. Moreover, the composition of the gut microbiota is influenced by multiple factors, including genetic background, dietary patterns, and geographic environment. Individuals with SUD often exhibit lifestyle disturbances, including altered dietary habits, increased psychological stress, comorbid conditions, and polysubstance abuse, which in turn significantly impact the diversity and functional profile of the gut microbiota. This makes it difficult to isolate the effects of addictive substances on gut microbiota composition in clinical studies.

Although interventions such as probiotics and FMT have shown certain regulatory effects and therapeutic potential in animal models, FMT faces substantial scientific and ethical barriers. Key limitations include that the safety and efficacy of FMT in treating addiction in humans remain unverified. Moreover, non-standardized donor screening protocols, uncontrolled transmission of viral communities, and the potential risk of antibiotic resistance gene transfer pose addressable yet critical issues for clinical FMT. Advancing research for addiction treatment via FMT critically depends on interlinked strategies. These include elucidating the mechanistic functions of specific microbial consortia, developing biobanked donor strain repositories with standardized preparation protocols, and ultimately creating synthetic microbial capsule therapeutics. As research in this field progresses, equal attention must be given to patient-centered care and ethical responsibility. In animal experimentation, the “3Rs” principle (Replacement, Reduction, and Refinement) should be rigorously applied to minimize suffering. In human studies, participants’ rights to informed consent, privacy, and autonomy must be protected, especially when working with vulnerable populations such as those with mental illness or substance dependence. Furthermore, enhanced ethical evaluation and social support systems are crucial.

## Conclusion

Based on existing evidence, the gut microbiota plays a predominant role in modulating substance abuse through several key mechanisms, including the regulation of metabolic and signaling processes by microbial metabolites, immune activation mediated by gut microbes, and neuroendocrine modulation via the HPA axis and VN. Preclinical and clinical studies highlight the potential of microbiota-targeted interventions, such as probiotics, prebiotics, FMT, and dietary modifications, in restoring gut homeostasis and enhancing addiction treatment outcomes. Future research should focus on elucidating the cellular and molecular mechanisms by which gut microbiota contribute to addiction and its comorbidities. Identifying key microbial metabolites, neurotransmitter precursors, and immune modulators will be essential in developing targeted therapies. Additionally, precision medicine approaches tailored to individual gut microbiota profiles could optimize treatment efficacy, given that genetics, diet, sex, and lifestyle significantly shape microbial composition.

## Abbreviations

5-HT: Serotonin
AMPs: Antimicrobial peptides
AhR: Aryl hydrocarbon receptor
AUD: Alcohol use disorder
BLA: Basolateral amygdala
BBB: Blood-brain barrier
BDNF: Brain-derived neurotrophic factor
CBD: Cannabidiol
CO: Carbon monoxide
CNA: Central nervous system
CeA: Central nucleus of the amygdala
CGA/ADRα2A: Chromogranin A/Adrenergic Receptor α2A
CEE: Chronic ethanol exposure
CPP: Conditioned place preference
CCH 200: Coral calcium hydride 200
CCL-11: Eotaxin-1
CPT-11: Irinotecan
e-cigs: Electronic cigarettes
EVs: Bacterial extracellular vesicles
FMT: Fecal microbiota transplantation
GABA: Gamma-aminobutyric acid
GABAergic: Gamma-aminobutyric acidergic
GBA: Gut-brain axis
G-CSF: Granulocyte colony-stimulating factor
GLP-1: Glucagon-like peptide-1
GI: Gastrointestinal
GPCRs: G protein-coupled receptors
HPA: Hypothalamic-pituitary-adrenal
IAA: Indole-3-acetic acid
IFN-γ: Interferon-γ
IL: Interleukin
iNOS: Nitric oxide synthase
IP: Intestinal permeability
IPA: Indole-3-pro-pionic acid
LPS: Lipopolysaccharide
LTA: Lipoteichoic acid
MCP-1: Monocyte chemoattractant protein-1
METH: Methamphetamine
n-3 PUFAs: n-3 polyunsaturated fatty acids
NAc: Nucleus accumbens
NAFLD: Non-alcoholic fatty liver disease
NF-κB: Nuclear factor kappa B
NMDA: N-methyl-D-aspartate
NTS: Nucleus tractus solitarius
PGN: Peptidoglycan
PXR: Pregnane X receptor
PYY: Peptide YY
Reg: Regenerating family
ROS: Reactive oxygen species
rTMS: Repetitive transcranial magnetic stimulation
SCFAs: Short chain fatty acids
SIGMAR1: Sigma-1 receptor
SNP: Single nucleotide polymorphism
SUD: Substance use disorder
TCA: Tricarboxylic acid
TGR5: G protein bile acid receptor
TLR: Toll-like receptor
TNF-α: Tumor necrosis factor-ɑ
Trp: Tryptophan
TRP/TPH-OR: Tryptophan hydroxylase-opioid receptor
TRKB: Tyrosine kinase B
VN: Vagus nerve
VTA: Ventral tegmental area
VOCs: Volatile organic compounds
ZO-1: Zonula occludens-1
